# Patients' Attitudes to Magnetic Resonance Imaging in Perianal Fistulizing Crohn’s Disease: A Global Survey

**DOI:** 10.1093/crocol/otaf015

**Published:** 2025-03-12

**Authors:** Easan Anand, Jalpa Devi, Anna Antoniou, Shivani Joshi, Jaap Stoker, Phillip Lung, Ailsa Hart, Phil Tozer, David H Ballard, Parakkal Deepak

**Affiliations:** Department of Surgery & Cancer, Imperial College London, London, UK; Robin Phillips Fistula Research Unit, St Mark’s the National Bowel Hospital, London, UK; Division of Gastroenterology, Washington University School of Medicine, St Louis, Missouri, USA; Department of Surgery & Cancer, Imperial College London, London, UK; Department of Surgery & Cancer, Imperial College London, London, UK; Robin Phillips Fistula Research Unit, St Mark’s the National Bowel Hospital, London, UK; Radiology and Nuclear Medicine, Amsterdam University Medical Centre, University of Amsterdam, The Netherlands; Robin Phillips Fistula Research Unit, St Mark’s the National Bowel Hospital, London, UK; Department of Surgery & Cancer, Imperial College London, London, UK; Robin Phillips Fistula Research Unit, St Mark’s the National Bowel Hospital, London, UK; Department of Surgery & Cancer, Imperial College London, London, UK; Robin Phillips Fistula Research Unit, St Mark’s the National Bowel Hospital, London, UK; Division of Gastroenterology, Washington University School of Medicine, St Louis, Missouri, USA; Division of Gastroenterology, Washington University School of Medicine, St Louis, Missouri, USA

**Keywords:** PPI, fistula, perianal Crohn’s disease, MRI, healing, radiology

## Abstract

**Background:**

There is limited patient involvement in radiological research for perianal fistulizing Crohn’s disease (pfCD), despite magnetic resonance imaging (MRI)’s critical role in diagnosis and management. Patient and public involvement is essential for aligning research with patient priorities. This study aimed to gather patient perspectives on the use of MRI in pfCD.

**Methods:**

A mixed-methods approach was used, following Guidance for Reporting Involvement of Patients and the Public (GRIPP2) guidelines. An online survey, co-developed with a patient representative, included open and closed questions on MRI experiences, advantages, challenges, and the potential for Artificial Intelligence (AI)-generated reports. This was followed by a virtual session for further exploration of patient views. Thematic analysis was conducted on the data.

**Results:**

Forty-seven patients with Crohn’s disease (37 with pfCD) from 6 countries participated, with 28/37 (76%) completing the survey. Key themes included patient expectations for MRI, preferences for scan intervals, and report content. Most (93%) wanted MRI reports to compare with previous scans, highlighting fistula changes and new abscesses. A majority (57%) preferred MRI scans annually when well, and more frequently after surgery (64.3% preferred scans at 3 months). Emotional relief was associated with MRI improvements, though access to services and report clarity remained challenging. Interest in AI-generated reports was expressed if clearly explained and validated by professionals.

**Conclusions:**

This is the first study exploring patient views on MRI use in pfCD, emphasizing the need for patient-centred MRI reporting and clearer communication. Future work should enhance patient access and validate AI-generated MRI reports.

## Graphical Abstract

**Graphical Abstract:**
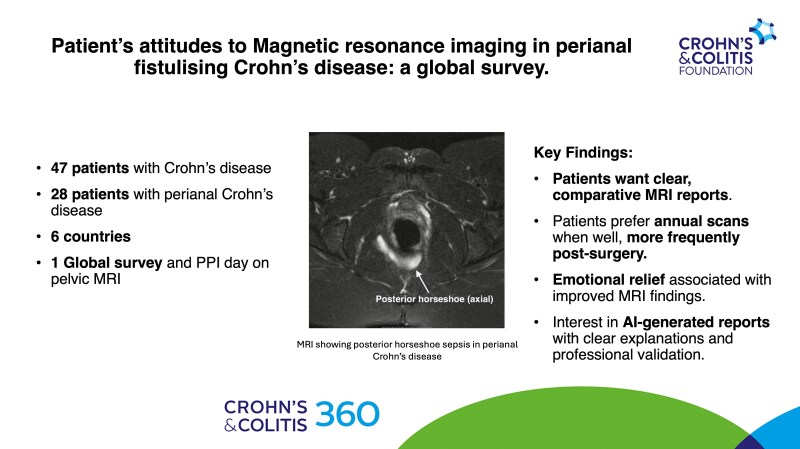

## Introduction

Perianal fistulizing Crohn’s disease (pfCD) affects approximately 20% of Crohn’s disease (CD) patients, particularly those with distal disease, and manifests as fistulae, abscesses, or other perianal symptoms.^1-3^ pfCD is linked to frequent relapses, impaired quality of life, high healthcare costs, and an increased risk of anorectal cancer,^4-6^ all of which contribute to repeated investigations. Perianal fistula-protocol pelvic magnetic resonance imaging (MRI) offers a safe, noninvasive method of disease assessment and is the preferred method for pre-operative assessment of fistulae.^7-9^ MRI-based radiological healing is associated with sustained fistula closure, as highlighted in the PISA II study^10^ and it is increasingly being used as an endpoint in clinical trials.^11^ Existing MRI index development and validation studies rarely incorporate patient perspectives.^12,13^ We lack a clear understanding of how radiological changes observed on MRI translate to meaningful impacts on a patient’s quality of life and the lack of patient engagement in these studies may hinder the alignment of imaging-based research with lived experiences.

Patient and public involvement (PPI) in research has gained substantial traction, with early initiatives in the UK dating back to the 1980s.^14^ PPI has demonstrated numerous benefits, including empowering patient input, fostering community engagement, and increasing patient enrollment in clinical trials.^15-18^ Despite these advancements, there remains a lack of comprehensive review regarding PPI in inflammatory bowel disease (IBD) research, even though IBD patients, who often actively manage their conditions, can offer valuable insights as experience-based experts.^19^ A systematic review by Al Khoury et al highlights the need to integrate patient perspectives into IBD care and research, emphasizing patients’ expectations for better disease education, shared decision-making, symptom control, and access to tools for effective communication and self-management.^20^

Recognizing this unmet need, we conducted an exploratory PPI study to engage patients and gather their perspectives on the use of MRI imaging in the context of pfCD. By incorporating patient insights into MRI research, this study aims to highlight areas for improvement in clinical practice, enhance patient-centred care, and contribute to the development of more effective frameworks for pfCD management.

## Materials and Methods

### Patient Recruitment and Demographics

This study adhered to the Guidance for Reporting Involvement of Patients and the Public (GRIPP2) Long Form checklist to ensure comprehensive and transparent reporting of PPI.^21^ The aim, methods, outcomes, reflections, and limitations of PPI have been systematically reported following GRIPP2 standards. In this study, we included one patient representative as a co-researcher (AA), consistent with the principles of the Patient-Oriented Research Level of Engagement Tool (PORLET).^22^ The representative contributed throughout the research process, including study design, data interpretation, and dissemination, ensuring meaningful engagement as outlined by PORLET.

Patients were recruited through IBD outpatient tertiary clinics, online social media including Twitter (X), Facebook, LinkedIn, and Instagram, as well as patient advocacy groups such as IBDesis and Crohn’s disease forums like Crohn Colitis UK (CCUK) and South Asian IBD Alliance (SAIA). The recruitment strategy targeted adults with pfCD. The broad recruitment method ensured that patients represented a diverse population in terms of geography and experiences with the disease within multiple healthcare settings.

### Survey Development and Distribution

A structured exploratory survey was developed to capture patient experiences and expectations regarding MRI usage in pfCD as part of PPI. The survey focused on key themes including patient expectations from MRI reporting, preferences for scan frequency, and interpretations of fistula healing. The survey was developed in collaboration with a patient representative from the study management group, ensuring alignment with both research goals and patient perspectives. This collaborative approach helped ensure the survey addressed the most relevant concerns and needs of the patient population. To address potential gaps in the survey, we included open-box questions throughout, a final section for additional feedback, and allowed for unstructured open discussion during the PPI session, with participants agreeing to be contacted for future study results and involvement.

Methods of dissemination included QR codes, allowing patients to scan and complete the survey on their phone, and links shared online on social media platforms, relevant IBD sites, and IBD clinics. This approach facilitated the collection of detailed insights into patients’ MRI experiences and preferences.

### Structure of the PPI Session

The PPI session was designed to gather in-depth patient feedback on their experiences and expectations regarding MRI imaging for pfCD. The session focused on four primary themes:

1. *Patient Expectations from MRI Reports:* Patients shared their views on the type of information they expect from MRI reports, emphasizing comparisons with previous scans and updates on the status of fistulae and abscesses.
2. *Frequency of MRI Scans:* This theme explored how often patients believe MRI scans should be performed for effective monitoring of their condition, especially following surgery or the initiation of new medical therapies.
3. *Definition of Healing on MRI:* Patients were asked to explain what they perceive as “improvement” or “healing” based on MRI results, considering both clinical factors (eg, reduction in fistula size) and emotional factors (eg, relief or hope).
4. *Interest in Artificial Intelligence (AI)-Generated MRI Patient—friendly summaries:* We also explored patients’ views on AI-generated MRI patient-friendly summaries, asking whether they would find such reports useful. Specifically, we inquired if they would want an AI-generated score to be included, how they would like this score to be explained, and whether they would appreciate comparative data from previous scans or actionable treatment recommendations from the AI if they were validated by a healthcare professional.

### Virtual PPI Day

Following the survey data collection, a virtual PPI day was organized involving 10 volunteer patients and a multidisciplinary team of experts, including gastroenterologists (IBD-ologists), radiologists, and colorectal surgeons around the globe. During the session, survey results were presented, and patients and experts discussed the findings and shared further experiences. This interactive forum allowed for an in-depth exploration of patients’ experiences with MRI and incorporated expert insights into the discussion, fostering a holistic understanding of the challenges and opportunities in using MRI for the management of pfCD.

### Data Collection and Analysis

Data were collected using 2 main methods: an online questionnaire and recordings from the PPI session. The questionnaire gathered quantitative demographic data and patient preferences regarding MRI reporting and frequency. The PPI session was recorded with the permission of patients and experts who participated, transcribed, and subjected to qualitative analysis.

For the qualitative analysis, thematic analysis was applied, following Braun and Clarke’s (2006) method.^23^ This process involved familiarization with the data, generating initial codes, and then categorizing the data into broader themes related to MRI use, reporting, and patient needs. This dual approach ensured that both quantitative trends and qualitative insights were captured, providing a comprehensive understanding of patient experiences with MRI scans for pfCD.

## Ethical Considerations

This study was conducted as part of a PPI exercise to inform and shape future research in radiology in pfCD and therefore the requirement for ethical approval was waived by the local review board. All patients received detailed information about the study and gave informed consent before participating in both the online questionnaire and the PPI session. To ensure confidentiality, all personal data were anonymized, and responses were securely stored in compliance with relevant data protection regulations. Patients were assured that their information would be kept confidential and used solely for research purposes.

## Results

### Patient Demographics

Of the 47 patients with Crohn’s disease who started the survey, 37 (79%) had pfCD, with 28/37 eligible patients (75.7%) completing the survey. The majority were female (93%), and the most common age group was 35-44 years (46.4%). 6 countries were represented during this exercise: patients were mostly from the UK (53.6%), followed by Canada (17.9%) and the USA (14.3%), with smaller numbers from Ireland (2), India (1) and Zimbabwe (1). 17 participants in total (including 10 patients and 7 clinicians) attended the virtual meeting (Table 1).

**Table 1.** Demographic characteristics of study participants.

Characteristic	*n* (%)
*Perianal Crohn’s disease*	
Total participants with Crohn’s disease	47
Participants with perianal Crohn’s disease	37 (79%)
Completed questionnaires	28/37 (75.7%)
Attended online PPI session (27.08.24)	17
*Country of residence*	
United Kingdom	15 (53.6%)
Canada	5 (17.9%)
United States	4 (14.3%)
Ireland	2 (7.1%)
India	1 (3.6%)
Zimbabwe	1 (3.6%)
*Gender*	
Female	26 (93%)
Male	2 (7%)
*Age group*	
18 to 24 years	1 (3.6%)
25 to 34 years	7 (25%)
35 to 44 years	13 (46.4%)
45 to 54 years	5 (17.9%)
55 to 64 years	2 (7.1%)

Percentages are based on the total number of eligible participants who completed the questionnaire (*N* = 28).

### Patient Perspectives on MRI Reports

**Figure 1.**
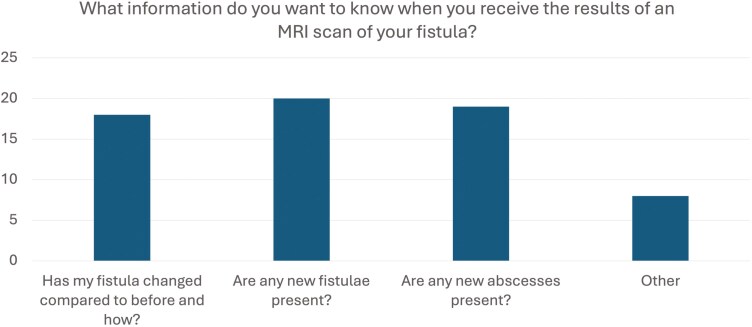
Key information patients seek from magnetic resonance imaging fistula scan reports.

Of pfCD patients who completed the survey, 26/28 (93%) as shown in **Figure 1** indicated that they wanted detailed information about changes in their fistula compared to previous MRI scans, including the nature and extent of such changes. Furthermore, 25/28 (89.3%) expressed interest in whether new fistulae had developed, while 24/28 (85.7%) emphasized the importance of identifying any new abscesses.

Patients also highlighted several other critical pieces of information that they wished to receive from their MRI reports. This included the precise location, position, and length of the fistula, as well as its associated activity within the body. Information about the presence of fluid in the original abscess area, the level of inflammation in surrounding tissues, and clarification regarding the type of fistula were also highly valued.

A. *Frequency of MRI Scans*

**Figure 2.**
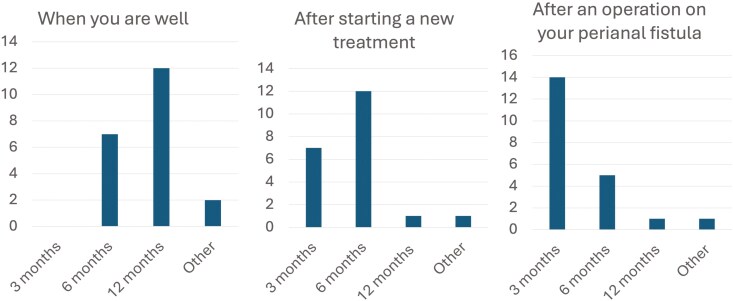
Preferred frequency of magnetic resonance imaging (MRI) Scans for perianal Crohn’s disease based on health status and treatment phase. *How often would you as a patient want to have MRI scans to assess your perianal Crohn’s disease?*

As shown in **Figure 2**. regarding the frequency of MRI scans, patients’ preferences varied depending on their disease status and treatment stage. When in remission, the majority, or 15/28 (53.6%) favoured scans every 12 months. However, after starting a new medical treatment, 15/28 (53.6%) of patients preferred scans after 6 months, whilst 8/28 (64.3 %) supported MRI scans every 3 months after surgical intervention. Patients “enjoyed” the regularity and noninvasive nature of MRI scans, particularly when compared to regular colonoscopies as part of their luminal investigations.

Key Quotation:

*“I actually enjoy the process of having an MRI scan, particularly when compared to a colonoscopy!”*

B. *Definitions of Improvement and Healing on MRI*

Patients’ perspectives on what constitutes “improvement” or “healing” on MRI scans revealed distinct themes related to physical, emotional, and treatment-related factors. Improvement was generally associated with a reduction in the size and length of the fistula tract, decreased inflammation, and the absence of new disease, which emotionally provided relief and hope.

Key Quotation:

*“It means the treatment plan I am currently on is working. It also means my quality of life should be improving”.*

**Figure 3.**
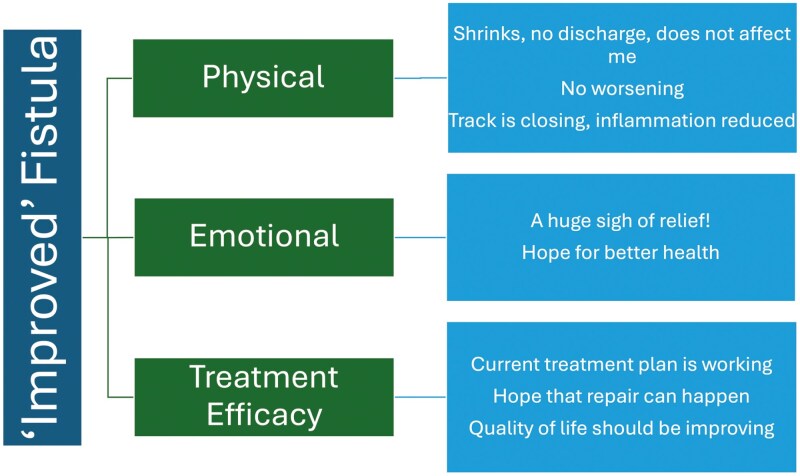
Dimensions of “improved” fistula in patients: physical, emotional, and treatment efficacy perspectives.

**Figure 4.**
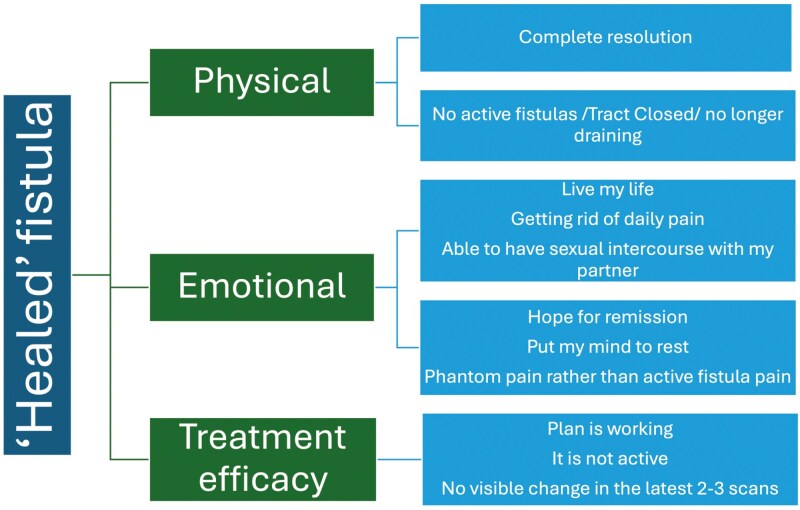
Dimensions of “healed” fistula in patients: physical, emotional, and treatment efficacy perspectives.

Complete healing, however, was characterized by the closure of the fistula tract on the MRI and no signs of active disease, although patients acknowledged that some scarring may remain. Healing was seen as a profound source of emotional relief, offering hope for long-term remission. (**Figures 3 and 4).**

Key Quotation:

*“Get back to being able to actually live my life, go on and not have to worry about anything, getting rid of daily pain that has been the norm for so many years. And being able to have sexual intercourse with my partner!!!”*

### Advantages and Challenges of MRI Scans

MRI scans were viewed favourably by patients, with many citing the noninvasive nature of the procedure as a key advantage. Patients appreciated the ability of MRI to assess fistula tracts, inflammation, and bowel wall thickening without the need for anesthesia or surgery. Many patients expressed trust in the accuracy of MRI scans for guiding treatment decisions, particularly in determining the effectiveness of current treatments and the necessity of future surgical interventions.

Key Quotation:

*“Peace of mind that treatment is working, much less invasive than other procedures, much easier than other procedures”.*

**Figure 5.**
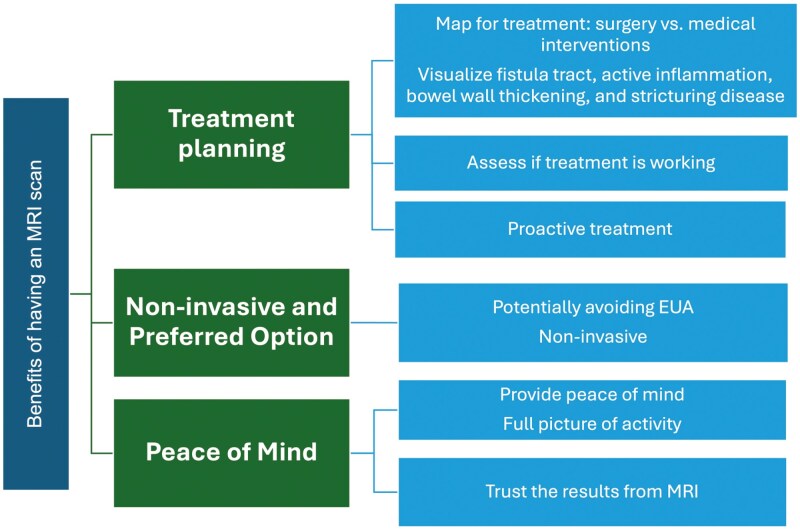
Key benefits of magnetic resonance imaging (MRI) scans in managing perianal fistulizing Crohn’s disease

**Figure 6.**
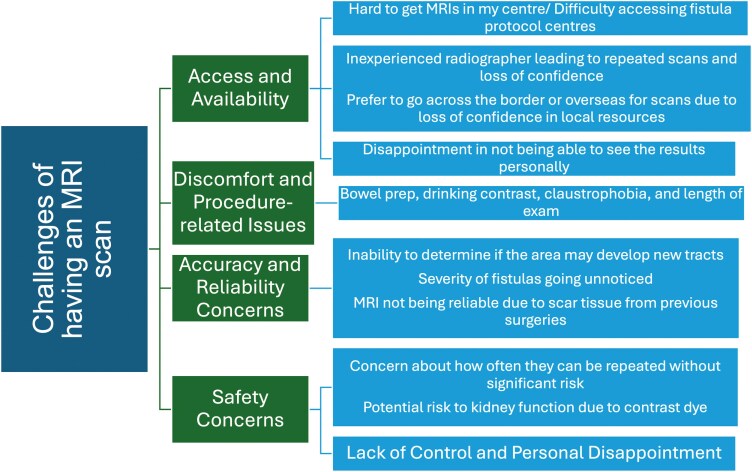
Challenges of magnetic resonance imaging scans for perianal fistulizing Crohn’s disease.

Despite the positive views on MRI, several challenges were identified. Access and availability of MRI appointments were reported as a significant issue, especially in centers with specialized fistula protocols. Some patients found MRI reports difficult to understand, describing them as “reading an unknown map.” Additionally, procedural discomfort, such as claustrophobia and the use of contrast agents, was a common concern (**Figures 5 and 6).** Some patients reported procedural discomfort during MRI scans, particularly those with claustrophobia or those required to endure lengthy exams. While no issues were reported with intravenous contrast, many patients noted discomfort with oral contrast agents, especially during combined abdominal and pelvic scans, expressing concerns about the process and potential impact on kidney function.

Key Quotation:

*“Not having access to a report causes stress and anxiety, which is often one of the causes of flares”.*

### Interest in AI-Generated MRI Patient-Friendly Summaries

**Figure 7.**
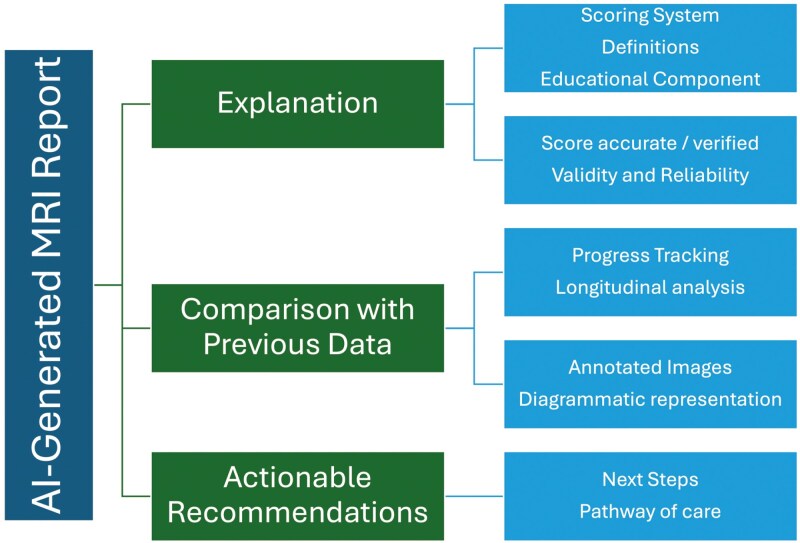
Key components of AI-generated patient-friendly summary of magnetic resonance imaging reports.

When asked about the potential use of AI-generated MRI patient-friendly summaries, patients expressed interest in receiving a clear explanation of the AI-generated score, including definitions and their significance in relation to their condition. They also favoured the inclusion of comparative data from previous MRI scans to track their disease progression. There was substantial interest in utilizing AI and 3D-rendered images of fistulas to enhance shared decision-making. Furthermore, patients expressed the need for actionable recommendations based on the report findings, including suggestions for future treatments and lifestyle modifications (**Figure 7**). Incorporating these insights, AI-generated MRI summaries could enhance patient empowerment by making complex medical information accessible, providing tools for better self-monitoring, and creating a foundation for informed discussions with clinicians. These features would align imaging-based diagnostics with patient priorities, ultimately promoting a more collaborative approach to care.

Key Quotation:

*“I’d want to understand what goes into that AI score, which features are used to score worst to ‘best’. Severity? Length of tracts, number of tracts, how much are in hard or soft tissue, how much in sphincter muscle”.*

## Discussion

This is the first study to date that specifically explores the views of pfCD patients on the use of MRI in the diagnosis and assessment of this challenging condition. Patients expressed a clear preference for MRI reports that offer detailed comparisons with previous scans (93% agreement), especially with regard to fistula changes, new abscesses, and fistula activity. These insights highlight the importance of ensuring that MRI reports are not only technically accurate but also accessible to patients, providing information that directly influences their treatment decisions. Previous research supports the notion that comprehensive and patient-friendly radiological reports can enhance patient involvement in their own care, leading to improved satisfaction and better health outcomes.^14,24^ Moreover, patients reported “enjoying” MRI scans and looking forward to the schedule of assessments. The consensus during the virtual meeting was that MRI scans were preferable to endoscopic assessment for luminal disease, were quick and relatively pain-free.

Historically, MRI reports have primarily served as clinical documents intended for clinician-to-clinician communication. However, there is now potential for a dual-purpose approach, with a separate component aimed at patient communication. This shift could enhance dialogue between patients and clinicians, demystifying the “black box” nature of the process. By making the information more accessible, patients who choose to engage can gain greater awareness of their condition, leading to increased involvement in their care.

Our findings align with current literature on the role of MRI in the management of pfCD, where MRI is regarded as the reference standard for assessing fistulas.^8^ However, the challenge remains to integrate patient needs into these assessments. The PPI session emphasized that patients want more than just clinical data; they want insights into how these findings will impact their treatment and quality of life. This need for actionable MRI reports mirrors the larger movement toward patient-centred care, where the delivery of health information is tailored to the patient’s specific condition and treatment trajectory.^16,17^

### Emotional Impact of MRI Findings on Patients

The emotional weight that patients attach to their MRI results cannot be overstated. For many patients, improvements in MRI findings—such as reduced fistula size or decreased inflammation—were closely linked to emotional relief and hope, signifying progress in their treatment. On the other hand, the idea of “healing” was often reserved for cases where fistulas had completely closed, though patients acknowledged the potential for scarring and relapse.

These emotional responses are consistent with the broader psychological impact that chronic diseases like Crohn’s disease exert on patients. Studies have shown that the perception of healing or improvement, as conveyed through imaging results, can dramatically affect a patient’s mental health and outlook on their condition.^18,19^ By engaging patients in discussions about their radiological results, healthcare providers can help mitigate anxiety and foster a stronger sense of partnership in the treatment process. Ensuring that MRI reports are not only clinically accurate but also explained in ways that provide emotional reassurance is critical to supporting the psychological well-being of patients with pfCD.^25^

### Challenges in MRI Accessibility and Report Comprehension

While the advantages of MRI in pfCD management are well-documented, patients in our study raised concerns about access to MRI services and the complexity of MRI reports. The limited availability of MRI appointments presents a barrier to timely diagnosis and treatment planning. Similar restrictions have been noted in other studies of IBD patients, where the demand for specialized imaging often exceeds available resources, especially in public healthcare systems.^26,27^

Additionally, the challenge of understanding MRI reports was a common theme. Many patients described MRI reports as difficult to interpret, equating the experience to “reading an unknown map.” This feedback points to a broader issue in healthcare: the need to make medical information more comprehensible for patients. Existing research underscores the value of providing patients with clear, layperson-accessible summaries of their imaging results, which can help bridge the gap between clinical language and patient understanding.^19^ Moreover, the development of simplified reporting formats, alongside professional consultation, could address this challenge and improve the utility of MRI findings for both patients and clinicians.

### Interest in AI-Generated Patient-Friendly Summaries of MRI Reports

A novel aspect of this study was the patients’ interest in AI-generated patient-friendly summaries of MRI reports. Patients were intrigued by the possibility of receiving AI-assisted assessments that could offer clear, objective scores on the severity and healing of fistulas, as well as comparisons with previous scans. However, patients were cautious and emphasized the importance of having these AI findings validated by medical professionals. There was substantial interest in leveraging large language models to generate patient-friendly summaries of medical information, incorporating actionable recommendations for both patients and clinicians. This approach could enhance patient understanding while providing clear guidance for clinical decision-making. LLM like ChatGPT have demonstrated potential in generating patient-friendly summaries of radiology reports by simplifying complex medical language while maintaining factual accuracy and improving readability.^28-30^ In the future, AI could be used to develop personalized 3D models by building on previous work performed using manual segmentation of perianal fistulae.^31,32^ These models could improve patient-clinician communication by providing clear, patient-specific visualizations. Additionally, linking patient-friendly MRI reports to actionable insights, such as medication recommendations or predictions about future disease progression and potential surgeries, would require further investigation and a substantial amount of longitudinal data to ensure accuracy and reliability. However, as our findings suggest, patient trust in AI technology hinges on its integration with traditional clinical judgment. Research indicates that when AI tools are used in conjunction with expert oversight, they can significantly enhance diagnostic accuracy and treatment planning.^33,34^

## Limitations of the Study

A PPI session on radiology and patient experiences of MRI in pfCD has several notable limitations. First, the inclusivity of non-English-speaking patients was limited, which could reduce the comprehensiveness of the analysis, particularly given the potential differences in healthcare systems. Furthermore, there was a Western bias in the respondents and discussion, as the panel consisted solely of individuals from Western healthcare systems. This could skew the perspectives and make the findings less applicable to other global contexts. The use of an online platform for recruitment may have limited participation to those with internet access, potentially skewing the demographic representation. The timing of the event was well-suited for a European and North American audience but may have been less convenient for those in Asia and East Asia due to time zone differences. Additionally, discussions were often limited by responder bias and the influence of dominant personalities, though efforts were made to mitigate this by incorporating an online survey alongside the virtual session. This study focused on MRI as the primary imaging modality and did not explore other diagnostic tools such as transperineal ultrasound (TPUS), which may offer a more accessible and patient-friendly alternative in certain settings. Future studies should investigate patient preferences regarding TPUS versus MRI, particularly in resource-limited environments and for patients with contraindications to MRI. While the broad nature of the topics covered prevented a deep dive into any single project, this approach helped to identify numerous potential areas for future research.

## Conclusion

This unique PPI session underscores the critical role of patient involvement in refining radiological research in pfCD. By incorporating patient feedback, particularly around MRI report content and frequency, researchers can improve the relevance of future endeavors, ensuring study aims remain patient-centred throughout. Addressing barriers to MRI accessibility and enhancing the clarity of reports are essential steps in making imaging a more patient-centred tool. As we move toward integrating AI technologies in radiology, maintaining a balance between innovation and patient trust will be key to achieving better outcomes. Incorporating AI in MRI reporting represents a promising avenue for enhancing both the accuracy and clarity of imaging results. Further research is needed to explore how AI can be tailored to provide patient-centred recommendations while being thoroughly validated by healthcare professionals.

## Data Availability

Data sharing is not applicable to this article as no patient datasets were generated or analyzed during the current study.

## Patient and Public Involvement

Patients were involved in the design phase of this research.
